# Von Hippel-Lindau regulates interleukin-32β stability in ovarian cancer cells

**DOI:** 10.18632/oncotarget.19311

**Published:** 2017-07-17

**Authors:** Hyo Jeong Yong, Jeong Su Park, Ae Lee Jeong, Sora Han, Sunyi Lee, Hye In Ka, Buyanravjkh Sumiyasuren, Hyun Jeong Joo, Su Jeong So, Ji Young Park, Do-Young Yoon, Jong-Seok Lim, Myeong-Seok Lee, Hee Gu Lee, Young Yang

**Affiliations:** ^1^ Department of Biological Sciences, Sookmyung Women’s University, Seoul, Republic of Korea; ^2^ Department of Severance Biomedical Science Institute, Yonsei Biomedical Research Institute, Yonsei University College of Medicine, Seoul, Korea; ^3^ Department of Bioscience and Biotechnology, Bio/Molecular Informatics Center, Konkuk University, Seoul, Republic of Korea; ^4^ Immunotherapy Convergence Research Center, Korea Research Institute of Bioscience and Biotechnology, Daejeon, Republic of Korea

**Keywords:** interleukin-32, von Hippel-Lindau, protein kinase C, hypoxia, apoptosis

## Abstract

Hypoxia-induced interleukin-32β (IL-32β) shifts the metabolic program to the enhanced glycolytic pathway. In the present study, the underlying mechanism by which hypoxia-induced IL-32β stability is regulated was investigated in ovarian cancer cells. IL-32β expression increased under hypoxic conditions in ovarian cancer cells as it did in breast cancer cells. The amount of IL-32β was regulated by post-translational control rather than by transcriptional activation. Under normoxic conditions, IL-32β was continuously eliminated through ubiquitin-dependent degradation by the von-Hippel Lindau (VHL) E3 ligase complex. Oxygen deficiency or reactive oxygen species (ROS) disrupted the interaction between IL-32β and VHL, leading to the accumulation of the cytokine. The fact that IL-32β is regulated by the energy-consuming ubiquitination system implies that it plays an important role in oxidative stress. We found that IL-32β reduced protein kinase Cδ (PKCδ)-induced apoptosis under oxidative stress. This implies that the hypoxia- and ROS-stabilized IL-32β contributes to sustain survival against PKCδ-induced apoptosis.

## INTRODUCTION

Interleukin-32 (IL-32) was originally identified as a secretory protein that enhances the production of pro-inflammatory cytokines, including IL-1, IL-6, and tumor necrosis factorα (TNF-α). Later, it was unveiled that IL-32 exists intracellularly in nonimmune cells such as epithelial and tumor cells [1–3]. IL-32 has nine isoforms that are generated by the alternative splicing of seven exons [4]. Functional studies of IL-32 in cancer cells have focused mainly on the IL-32α, IL-32β, and IL-32γ isoforms because of their abundance [5]. IL-32α and IL-32β are highly expressed in gastric, lung, pancreatic, breast, brain, and liver tumors [6–8], but they show context-dependent function in different tumors. For example, IL-32α shows anti-proliferative effects in human colon cancer cells [9–11], whereas it enhances migration in human melanoma cells through the downregulation of E-cadherin [12] and promotes proliferation in pancreatic cells [6]. In addition, IL-32β is positively correlated with tumor size, number of lymph node metastases and tumor stage in primary breast cancer tissues; increases migration and invasion through signal transducer and activator of transcription 3 (STAT3) activation; and enhances glycolysis in human breast cancer cells [13, 14]. On the other hand, it shows anti-tumor activity through the activation of lymphocytes including cytotoxic T (CD8+) and natural killer (NK) cells, and inactivation of the nuclear factor-κB (NF-κB) and STAT3 signaling pathways [15].

The von Hippel-Lindau (VHL) tumor suppressor protein is an E3 ligase that ubiquitinates hypoxia-inducible factor-1α (HIF-1α) and causes its degradation by the proteasome [16, 17]. When oxygen is abundant, VHL binds to HIF-1α by recognizing its two post-translationally hydroxylated proline residues (P402 and P564) [18]. On the other hand, a lack of oxygen due to the rapidly growing tumor mass cause the inactivation of prolyl hydroxylase (PHD) preventing VHL from recognizing and binding to HIF-1α and thus leading to HIF-1α accumulation, which induces the transcription of genes involved in adaptation to hypoxia [19, 20]. In addition to prolyl hydroxylation, asparaginyl hydroxylation has also been identified as an oxygen-regulated signal that determines the stability of HIF-1α. Both reactions are catalyzed by members of the 2-oxoglutarate (2OG)-dependent oxygenase superfamily: HIF-1α prolyl hydroxylation by PHD domains 1–3, and HIF-1α asparaginyl hydroxylation by factor inhibiting HIF-1 α (FIH). Since hydroxylation is one of the important post-translational modifications, the assignment of molecular functions for all human 2OG oxygenases by combined biochemical and cellular approaches is required. As an effort, accumulating data on newly identified hydroxylated proteins are expanding [21, 22].

The protein kinase C (PKC) family comprises serine/threonine kinases that regulate a diverse set of cellular processes including proliferation, apoptosis, survival, and migration, and there is a substantial amount of evidence linking PKC to tumorigenesis [23]. According to their domain structure and respective activators, they have been divided into three major groups: the classical PKCs, including the α, βI, βII, and γ isoforms; the novel PKCs, including the θ, η, ε, and δ isoforms; and the atypical PKCs, including ζ and ι/λ [24]. Among various isoforms of PKC, PKCε and PKCδ have opposing roles in regulating apoptosis, survival and proliferation. PKCε promotes cell survival in many cell types, whereas PKCδ performs a growth inhibitory or pro-apoptotic role [23, 24]. When PKCδ is activated by oxidative and hypoxic stress, PKCδ forms complex with Abl in the endoplasmic reticulum (ER), and then PKCδ-Abl complex translocates from ER to the mitochondria, leading to caspase-3 cleavage and cytochrome c release to trigger apoptosis [24–26]. The functional connection between IL-32β and PKCδ has recently been unveiled by the report that IL-32β upregulates IL-10 production through its association with PKCδ around the nuclear membrane [27, 28].

In the present study, we investigated the function of IL-32β in human ovarian cancer cells which is one of the representative female cancers. Our results revealed that IL-32β is degraded by VHL-mediated ubiquitination, a process that is prevented by hypoxia-induced reactive oxygen species (ROS) production. PKCδ forms trimers with IL-32β and VHL. Under hypoxic conditions, the interaction between IL-32β and PKCδ is sustained, whereas that with VHL is disrupted. In turn, the increased IL-32β level reduces PKCδ-mediated apoptosis under oxidative stress.

## RESULTS

### IL-32β level increases under hypoxic conditions in ovarian cancer cells

**Figure 1:**
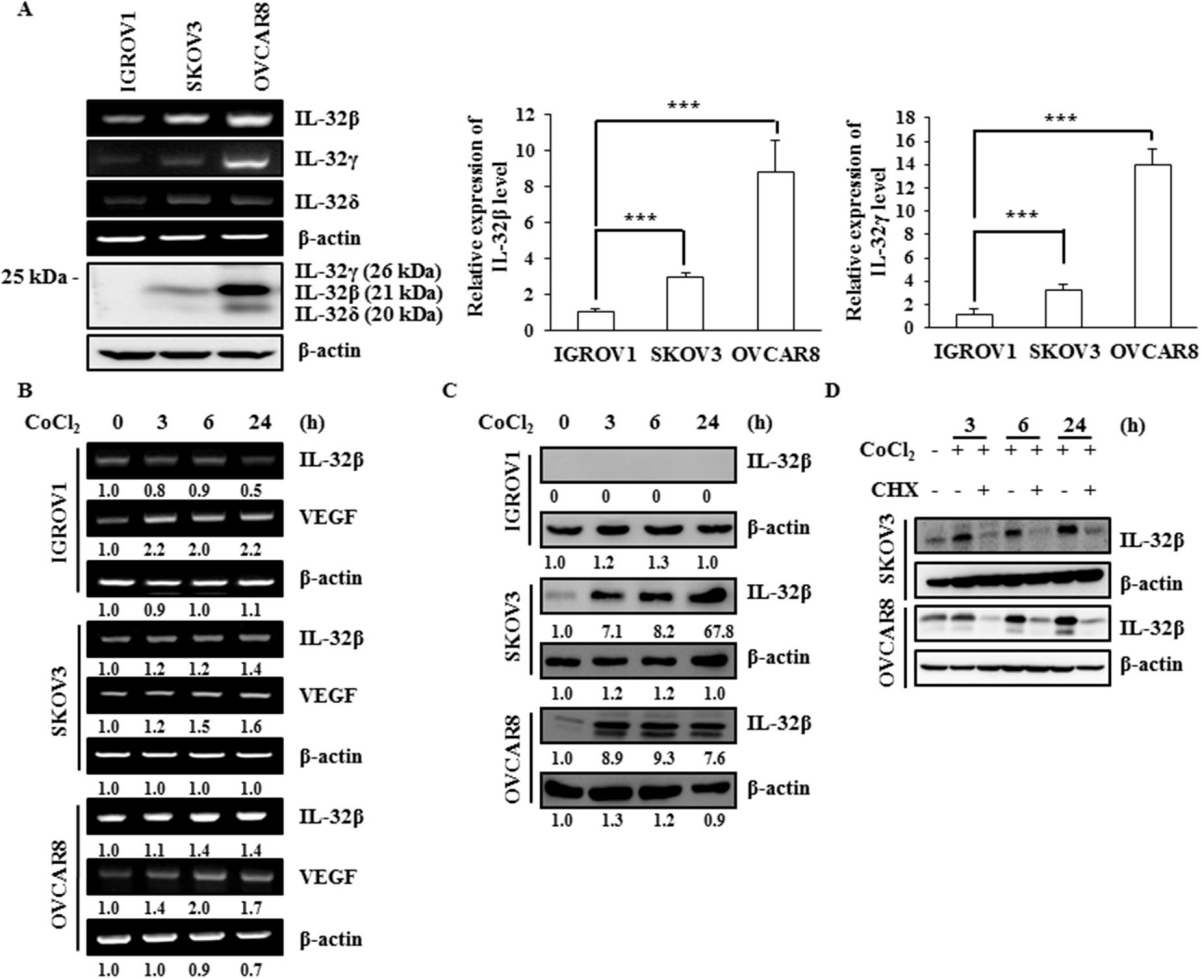
IL-32β levels increase under hypoxic conditions in ovarian cancer cells **(A)** The IL-32 levels in the human ovarian cancer cell lines IGROV1, SKOV3, and OVCAR8 were determined by RT-PCR, qRT-PCR, and immunoblot assays. The qRT-PCR results were normalized with the level of IL-32β and IL-32γ in IGROV1 cells, respectively. The data shown represent one of three independent experiments carried out in triplicate. **(B, C)** SKOV3 and OVCAR8 cells were treated with 150 μM CoCl_2_ and the IL-32β levels were determined by RT-PCR and immunoblot assays at the indicated times. The bands were quantified using ImageJ software and the numbers indicate the comparison of each lane. **(D)** SKOV3 and OVCAR8 cells were treated with 150 μM CoCl_2_ and 5 μg/mL CHX. The IL-32β levels were determined by immunoblot assay at the indicated times.

We had previously shown that IL-32β level is increased and enhances glycolysis in breast cancer cells to survive under hypoxic conditions [14]. In the present study, we determined the molecular mechanism by which IL-32β stability is increased under hypoxic conditions in ovarian cancer cells. The levels of IL-32 protein and mRNA were examined in three different human ovarian cancer cell lines: IGROV1, SKOV3, and OVCAR8 cells. Although IGROV1 cells expressed both IL-32β and IL-32γ mRNA, the proteins were not detected, even with longer exposure. SKOV3 cells showed lower levels of IL-32β and IL-32γ mRNA expression than OVCAR8 cells. On the other hand, the IL-32β and IL-32δ proteins were highly expressed in OVCAR8 cells (Figure 1A). To determine whether IL-32β expression is increased by hypoxic conditions, the ovarian cancer cells were treated with CoCl_2_, a hypoxia mimetic chemical. Whereas vascular endothelial growth factor (VEGF) mRNA expression (as a positive control) was markedly increased in all three cell lines, IL-32β mRNA expression was only modestly increased in SKOV3 and OVCAR8 cells (Figure 1B). However, the IL-32β protein was highly increased in SKOV3 and OVCAR8 cells 3 h after CoCl_2_ treatment, but IL-32β proteins were not detected even in the ROS-stimulated in IGROV1 cells (Figure 1C). One possibility is that IGROV1 cells may have epigenetically suppressed IL-32β. Thus, we determined whether IL-32β translation was increased. When cells were co-treated with cyclohexamide (CHX) and CoCl_2_ for the indicated time intervals, the CoCl_2_-induced increase in IL-32β level was not observed (Figure 1D). If IL-32β is regulated by proteasomal degradation, the pre-existing IL-32β should be detected after the CHX treatment. However, if the steady state level of IL-32β is very low, it is possible that IL-32β level is low in the presence of CHX.

### Hypoxic conditions inhibit IL-32β degradation

**Figure 2:**
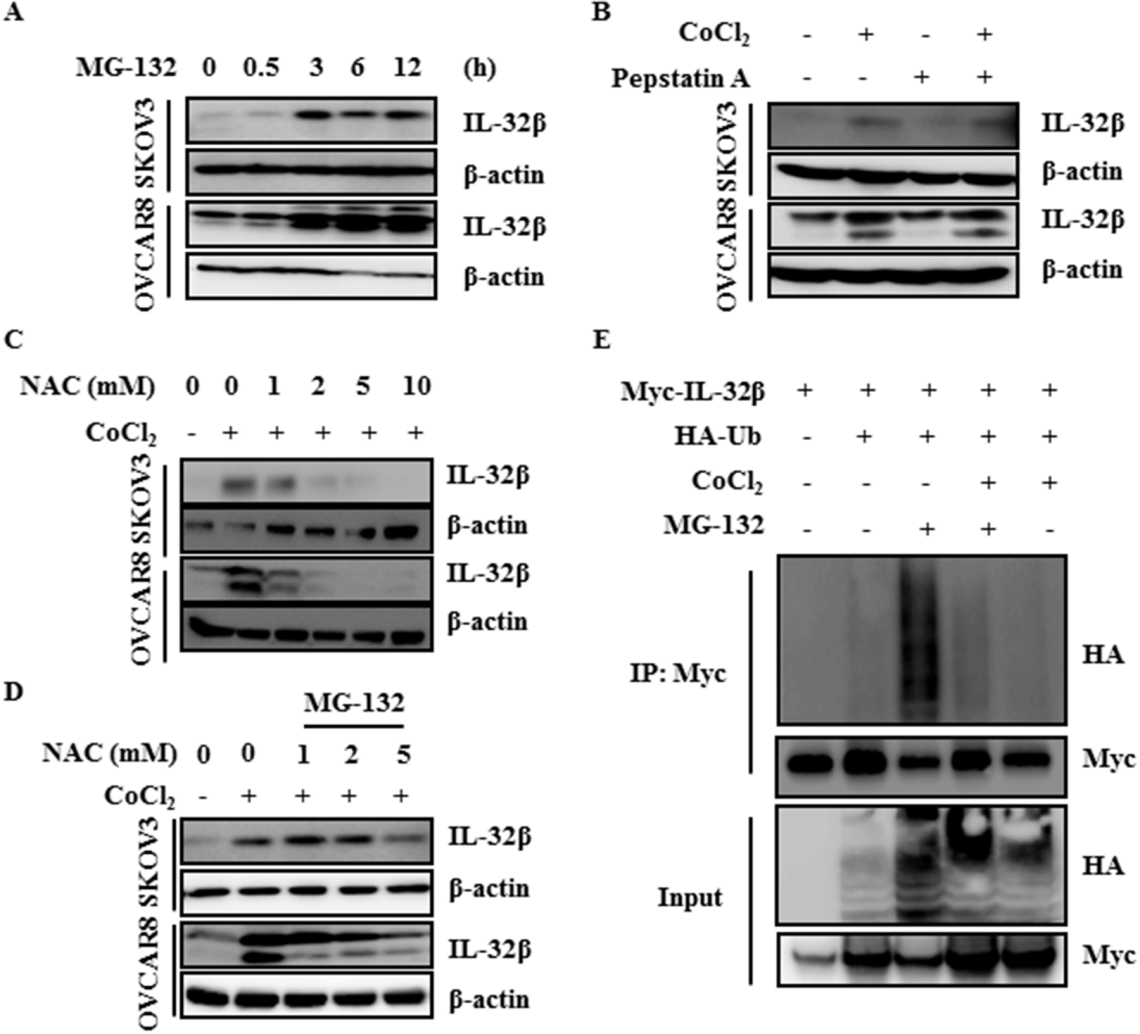
ROS inhibit ubiquitin-dependent IL-32β degradation **(A)** SKOV3 and OVCAR8 cells were treated with 5 μM MG-132 and the IL-32β levels were determined by immunoblot assay at the indicated times. **(B)** SKOV3 and OVCAR8 cells were pretreated with 1 μg/mL pepstatin A for 1 h, and then treated with 150 μM CoCl_2_. **(C)** SKOV3 and OVCAR8 cells were pretreated with NAC for 1 h, and then treated with 150 μM CoCl_2_. **(D)** SKOV3 and OVCAR8 cells were pretreated with NAC for 1h, and then treated with 150 μM CoCl_2_ and MG-132. **(E)** HEK 293T cells were transfected with Myc-IL-32β and HA-Ub, and then treated with 150 μM CoCl_2_ or 5 μM MG-132. The cell lysates were immunoprecipitated with anti-Myc antibody and the interaction was examined by immunoblot assay.

There are two mechanisms to explain the increase of protein amount: increase of the translation rate, and inhibition of protein degradation. To examine the possibility of proteasomal degradation, SKOV3 and OVCAR8 cells were treated with MG-132 for the indicated time intervals. IL-32β was clearly increased 3 h after the treatment (Figure 2A). However, when cells were treated with pepstatin A, an inhibitor of lysosomal degradation, the increase in IL-32β level was not observed (Figure 2B). These results imply that the translated IL-32β had been continuously degraded by ubiquitination. In the previous study, we showed that the CoCl_2_-induced increase in IL-32β level was mediated by ROS production in breast cancer cells. The ovarian cancer cells also showed the CoCl_2_-mediated increase in IL-32β production, which was prevented by treatment with N-acetyl-cysteine (NAC), an anti-ROS reagent, in a dose-dependent manner (Figure 2C), and co-treatment of MG-132 with NAC reverse the latter’s effect (Figure 2D). This implies that ROS stabilize IL-32β levels through the inhibition of proteasomal degradation. To further confirm that IL-32β is continuously degraded under normal conditions, we examined whether it is ubiquitinated. Cells were transfected with Myc-IL-32β and Hemagglutinin (HA)-ubiquitin (Ub) and then treated with CoCl_2_ or MG-132. IL-32β was immunoprecipitated with anti-Myc antibody and immunoblotted with anti-HA antibody to examine whether IL-32β is ubiquitinated. MG-132 treatment increased the level of ubiquitinated IL-32β, whereas CoCl_2_ treatment inhibited it (Figure 2E). Collectively, these results imply that hypoxia-induced ROS production increases IL-32β levels by preventing its ubiquitination.

### The VHL E3 ligase complex is responsible for IL-32β degradation

**Figure 3:**
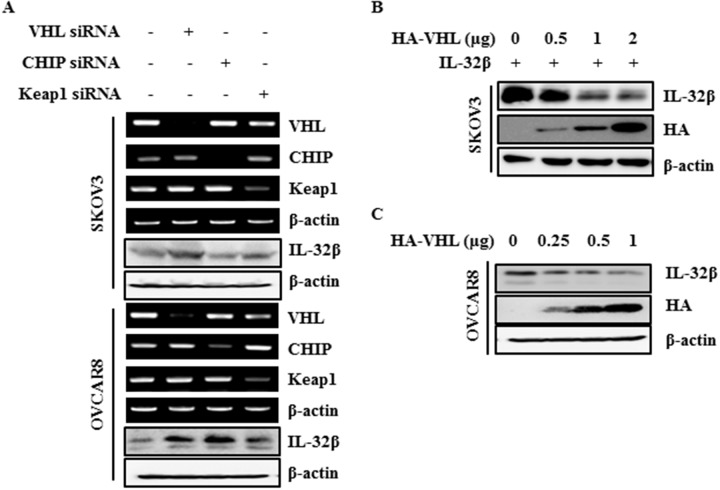
IL-32β is regulated by E3 ligase VHL **(A)** SKOV3 and OVCAR8 cells were transfected with VHL siRNA, CHIP siRNA, and Keap1 siRNA. The VHL, CHIP and Keap1 levels were determined by RT-PCR. The IL-32β levels were determined by immunoblot assay. **(B)** SKOV3 cells were co-transfected with IL-32β and HA-VHL-expressing plasmids. The IL-32β and VHL levels were determined by immunoblot assay. **(C)** OVCAR8 cells were transfected with HA-VHL. The IL-32β and VHL levels were determined by immunoblot assay.

VHL is a well-known E3 ligase involved in HIF-1α degradation under normoxic conditions. Kelch-like ECH-associated protein 1 (Keap1) is also an E3 ligase involved in nuclear factor erythroid 2-related factor 2 (Nrf-2) degradation in the absence of ROS, and Carboxyl terminus of HSP70-interacting protein (CHIP) is a chaperone-dependent E3 ligase that ubiquitylates unfolded protein under ROS stress [29–32]. To find out which E3 ligase is responsible for IL-32β ubiquitination, SKOV3 and OVCAR8 cells were transfected with VHL small interfering RNA (siRNA), CHIP siRNA, and Keap1 siRNA. The IL-32β was increased after VHL siRNA transfection (Figure 3A). To further confirm this, SKOV3 cells were induced to express IL-32β and HA-VHL, whereupon the IL-32β levels were decreased by HA-VHL in a dose-dependent manner (Figure 3B). Next, OVCAR8 cells highly expressing IL-32β were transfected with increasing amounts of HA-VHL, whereupon the endogenous IL-32β decreased (Figure 3C). These results imply that VHL is the E3 ligase responsible for IL-32β ubiquitination.

### ROS disrupt the interaction between VHL and the exon 7 coding region of IL-32β

**Figure 4:**
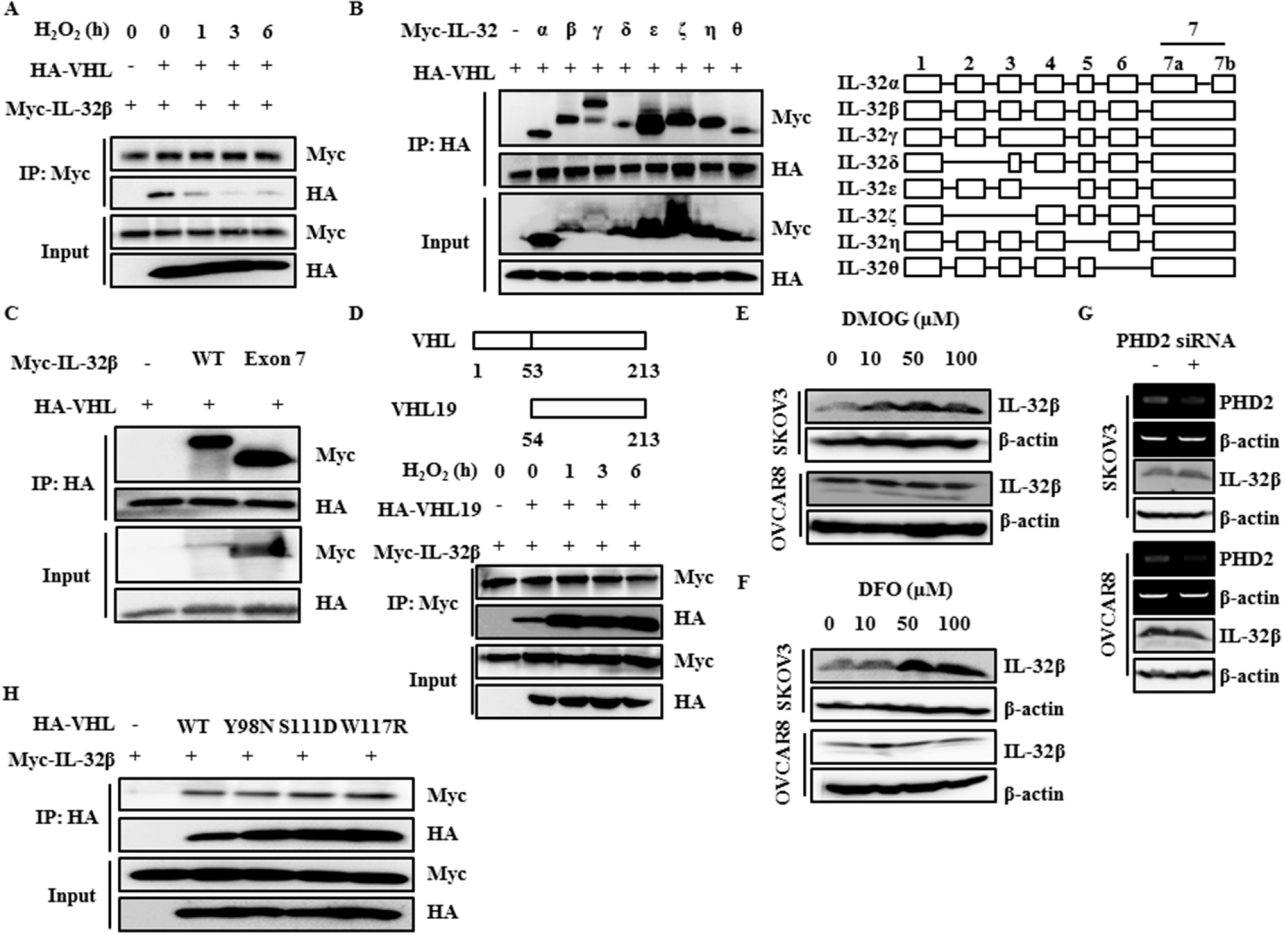
The interaction between IL-32β and VHL is disrupted by ROS **(A)** HEK 293T cells were transfected with Myc-IL-32β and HA-VHL, and then treated with 100 μM H_2_O_2_ for the indicated times. The cell lysates were immunoprecipitated with anti-Myc antibody and the interaction was examined by immunoblot assay. **(B)** HEK 293T cells were transfected with the Myc-IL-32 isoforms and HA-VHL. The cell lysates were immunoprecipitated with anti-HA antibody and the interaction was examined by immunoblot assay. **(C)** HEK 293T cells were transfected with Myc-IL-32β, Myc-IL-32 exon 7, and HA-VHL. The cell lysates were immunoprecipitated with anti-HA antibody and the interaction was examined by immunoblot assay. **(D)** HEK 293T cells were transfected with Myc-IL-32β and HA-VHL19, and then treated with 100 μM H_2_O_2_ for the indicated times. The cell lysates were immunoprecipitated with anti-Myc antibody and the interaction was examined by immunoblot assay. **(E)** SKOV3 and OVCAR8 cells were treated with DMOG for 24 h. The IL-32β levels were determined by immunoblot assay. **(F)** SKOV3 and OVCAR8 cells were treated with DFO for 24 h. The IL-32β levels were determined by immunoblot assay. **(G)** SKOV3 and OVCAR8 cells were transfected with PHD2 siRNA. The PHD2 levels were determined by RT-PCR. The IL-32β levels were determined by immunoblot assay. **(H)** HEK 293T cells were transfected with Myc-IL-32β, HA-VHL wild type (WT), or HA-VHL mutant. The cell lysates were immunoprecipitated with anti-HA antibody and the interaction was examined by immunoblot assay.

To investigate the underlying molecular mechanisms by which IL-32β is regulated by VHL E3 ligase, cells were induced to express Myc-IL-32β and HA-VHL and then treated with H_2_O_2_. The immunoprecipitation assay revealed that the VHL and IL-32β interaction was disrupted after H_2_O_2_ treatment (Figure 4A), implying that IL-32β is degraded by VHL-mediated ubiquitination and this degradation is prevented by hypoxia-induced ROS production. To determine which domain of IL-32 binds to VHL, the interaction between different IL-32 isoforms and VHL was examined. All isoforms of IL-32 [33] bound to VHL (Figure 4B). Since only the exon 7-coded region is common to all isoforms of IL-32, it was the only regions shown to interact with VHL (Figure 4C). To determine which domain of VHL binds to IL-32β, the interaction between IL-32β and VHL domain using VHL19, which is absent in the acidic domain of first 53 amino acid residues [34], was examined. The interaction between IL-32β and VHL19 was increased after H_2_O_2_ treatment, implying that acidic domain of VHL may play an important role in disruption of the interaction between IL-32β and VHL under hypoxic conditions (Figure 4D).

Next, we examined whether a proline residue of IL-32β is hydroxylated by PHD, since VHL binds only to such proline residues in HIF-1α [19, 20]. SKOV3 and OVCAR8 cells were treated with a PHD inhibitor, dimethyloxaloylglycine (DMOG) or desferrioxamine (DFO), and IL-32β expression was measured. Contrary to our expectation, DMOG and DFO did not affect the IL-32β level in OVCAR8 cells, although SKOV3 cells showed increase in IL-32β level (Figure 4E, 4F). In addition, SKOV3 and OVCAR8 cells were also transfected with PHD2 siRNA and the IL-32β was examined. No difference was observed (Figure 4G). To further confirm this result, we examined the interaction of IL-32β with VHL mutants that are not able to bind to hydroxyl proline residues of HIF-1α [19, 34]. All VHL mutants still bound to IL-32β (Figure 4H), implying that the interaction between IL-32β and VHL is not dependent upon proline hydroxylation.

### PKCδ is associated with the interaction between IL-32β and VHL

**Figure 5:**
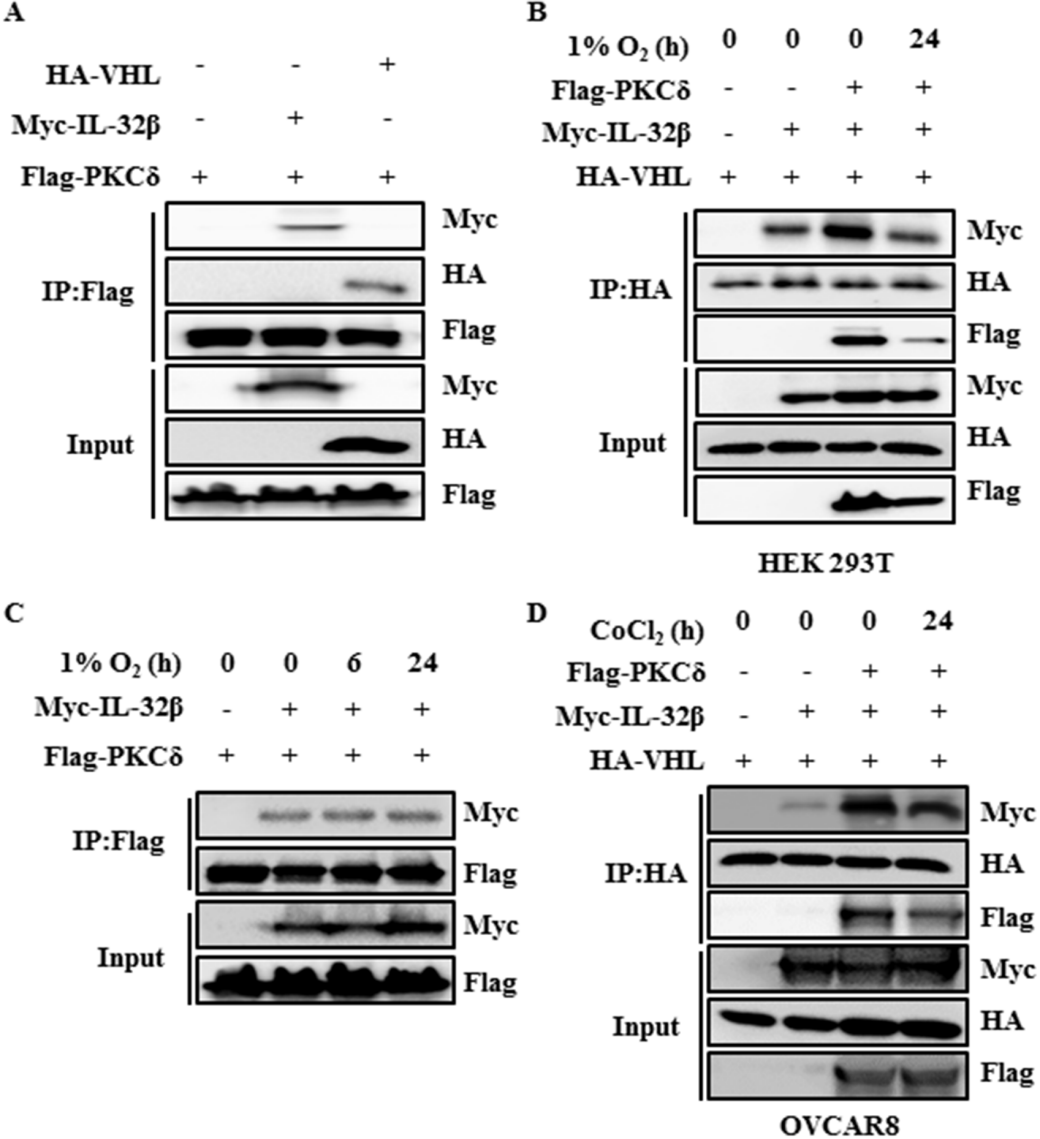
PKCδ forms a complex with IL-32β and VHL **(A)** HEK 293T cells were transfected with Myc-IL-32β, HA-VHL, and Flag-PKC. The cell lysates were immunoprecipitated with anti-Flag antibody and the interaction was examined by immunoblot assay. **(B)** HEK 293T cells were transfected with Myc-IL-32β, HA-VHL, and Flag-PKCδ, and then placed in a hypoxia chamber for 24 h. The cell lysates were immunoprecipitated with anti-HA antibody and the interaction was examined by immunoblot assay. **(C)** HEK 293T cells were transfected with Myc-IL-32β and Flag-PKCδ, and then placed in a hypoxia chamber for 24 h. The cell lysates were immunoprecipitated with anti-Flag antibody and the interaction was examined by immunoblot assay. **(D)** OVCAR8 cells were transfected with Myc-IL-32β, HA-VHL and Flag-PKCδ, and then treated with 150 μM CoCl_2_ for 24 h. The cell lysates were immunoprecipitated with anti-HA antibody and the interaction was examined by immunoblot assay.

Since it is known that ROS activates PKC, which can interact with IL-32 and VHL [27, 35–37], PKC was examined for its possible association with the interaction between IL-32β and VHL. Cells were induced to express HA-VHL, Myc-IL-32β and Flag-PKCδ, and then immunoprecipitated with anti-Flag antibody. PKCδ precipitated with Myc-IL-32β and HA-VHL (Figure 5A). In addition, when cells were placed in the hypoxic chamber, the co-immunoprecipitation of IL-32β and PKCδ with VHL was reduced (Figure 5B). These results imply that the interaction of VHL with either IL-32β or PKCδ is reduced under hypoxia. However, the interaction between IL-32β and PKCδ was constant regardless of the oxygen conditions (Figure 5C). To further confirm this interaction in human ovarian cancer cells, OVCAR8 cells were induced to express HA-VHL, Myc-IL-32β and Flag-PKCδ, and then immunoprecipitated with anti-HA antibody. VHL precipitated with Myc-IL-32β and Flag-PKCδ. In addition, when cells were treated with CoCl_2_, the co-immunoprecipitation of IL-32β and PKCδ with VHL was reduced (Figure 5D). These results collectively suggest that IL-32β, VHL, and PKCδ form a complex. Under the hypoxic condition, the interaction between VHL and IL-32β or PKCδ is disrupted, not affecting the interaction between IL-32β and PKCδ.

### IL-32β attenuates PKCδ-induced apoptosis in oxidative stress

**Figure 6:**
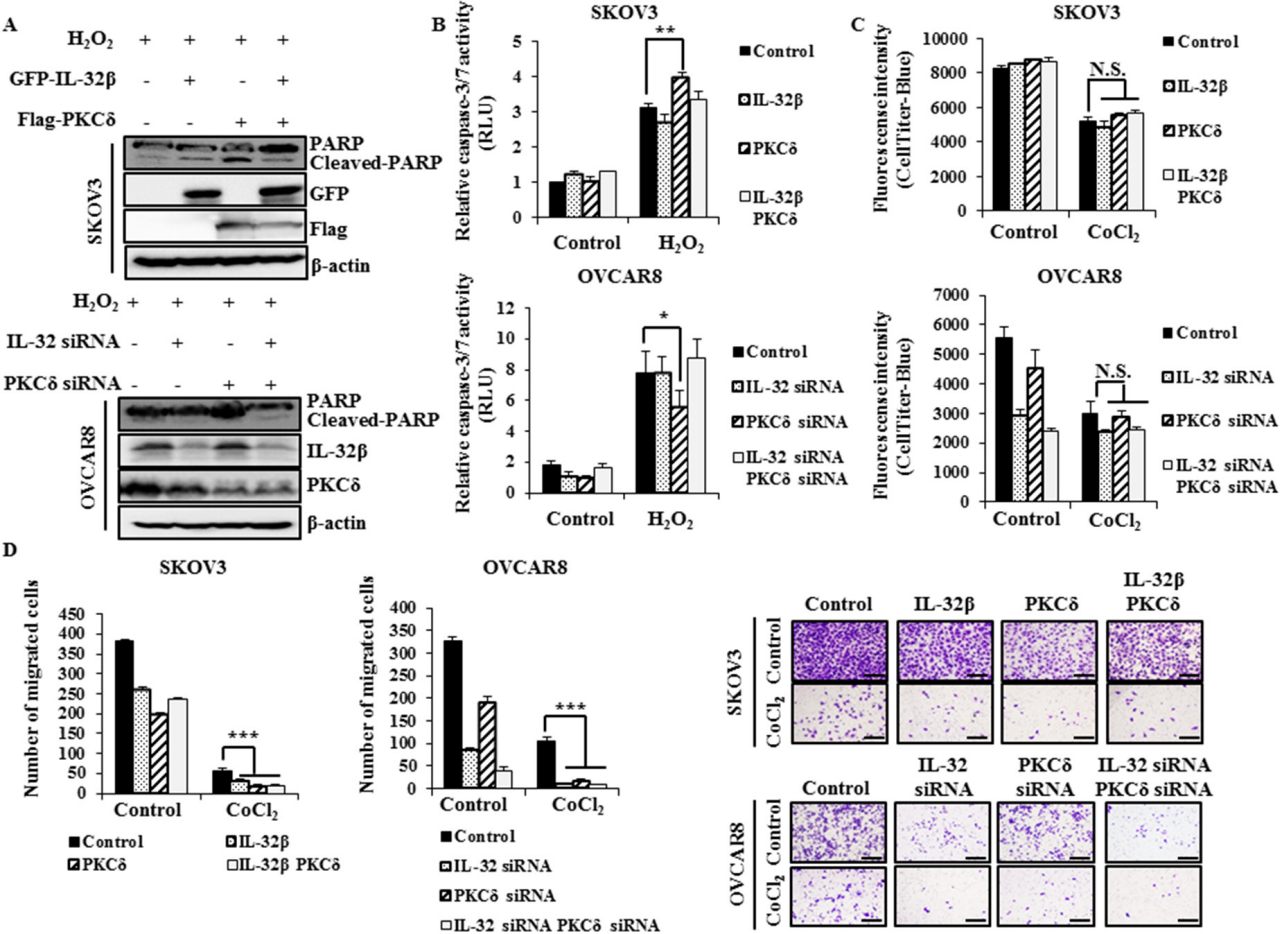
IL-32β prevents PKCδ-induced apoptosis under oxidative stress **(A, B)** SKOV3 cells were transfected with GFP-IL-32β and Flag-PKCδ for 24 h, whereas OVCAR8 cells were transfected with IL-32 siRNA and PKCδ siRNA for 48 h. The transfected cells were treated with 100 μM H_2_O_2_ for 24 h. The caspase-3/7 activity was assessed using a luminescent assay. The cleaved-PARP levels were determined by immunoblot assay. **(C)** SKOV3 cells were transfected with GFP-IL-32β and Flag-PKCδ. OVCAR8 cells were transfected with IL-32 siRNA and PKCδ siRNA, and then treated with 150 μM CoCl_2_ for 24 h. The proliferation was assessed using CellTiter-Blue. **(D)** SKOV3 cells were transfected with GFP-IL-32β and Flag-PKCδ, whereas OVCAR8 cells were transfected with IL-32 siRNA and PKCδ siRNA. The transfected cells were seeded onto the upper part of a Transwell chamber in serum-free medium with or without 150 μM CoCl_2_. The migrated SKOV3 and OVCAR8 cells were counted after 24 or 48 h incubation, respectively. Scale bars = 10 μm.

To find out effect of IL-32β and PKCδ co-expression on apoptosis, proliferation, and migration, SKOV3 cells expressing a low level of IL-32β were induced to express IL-32β and PKCδ, and OVCAR8 cells expressing a high level of IL-32β were transfected with IL-32 siRNA and PKCδ siRNA, and then cell apoptosis was examined since PKCδ is known to be involved in those functions [23]. PKCδ increased the level of cleaved-poly (ADP-ribose) polymerase (PARP) and the activity of caspase-3/7 after H_2_O_2_ treatment. However, co-expression of IL-32β and PKCδ reduced the H_2_O_2_-induced apoptosis in the presence of PKCδ, implying that IL-32β attenuates PKCδ-induced apoptosis under oxidative stress (Figure 6A, 6B). Since PKCδ also has an anti-proliferative effect on cancer cells, its effect was examined in the presence of IL-32β. The proliferation of SKOV3 and OVCAR8 cells was greatly reduced after CoCl_2_ treatment, but this was not affected by the co-presence of IL-32β and PKCδ (Figure 6C). Next, we examined effect of IL-32β and PKCδ on migration under CoCl_2_-induced hypoxic conditions. SKOV3 and OVCAR8 cell migration was considerably decreased after CoCl_2_ treatment, and this effect was not affected by the co-expression of IL-32β and PKCδ (Figure 6D). These findings imply that the accumulated IL-32β by hypoxia-induced ROS binds to PKCδ, leads to inhibit PKCδ-induced apoptosis under oxidative stress without affecting other functions, including proliferation and migration.

## DISCUSSION

**Figure 7:**
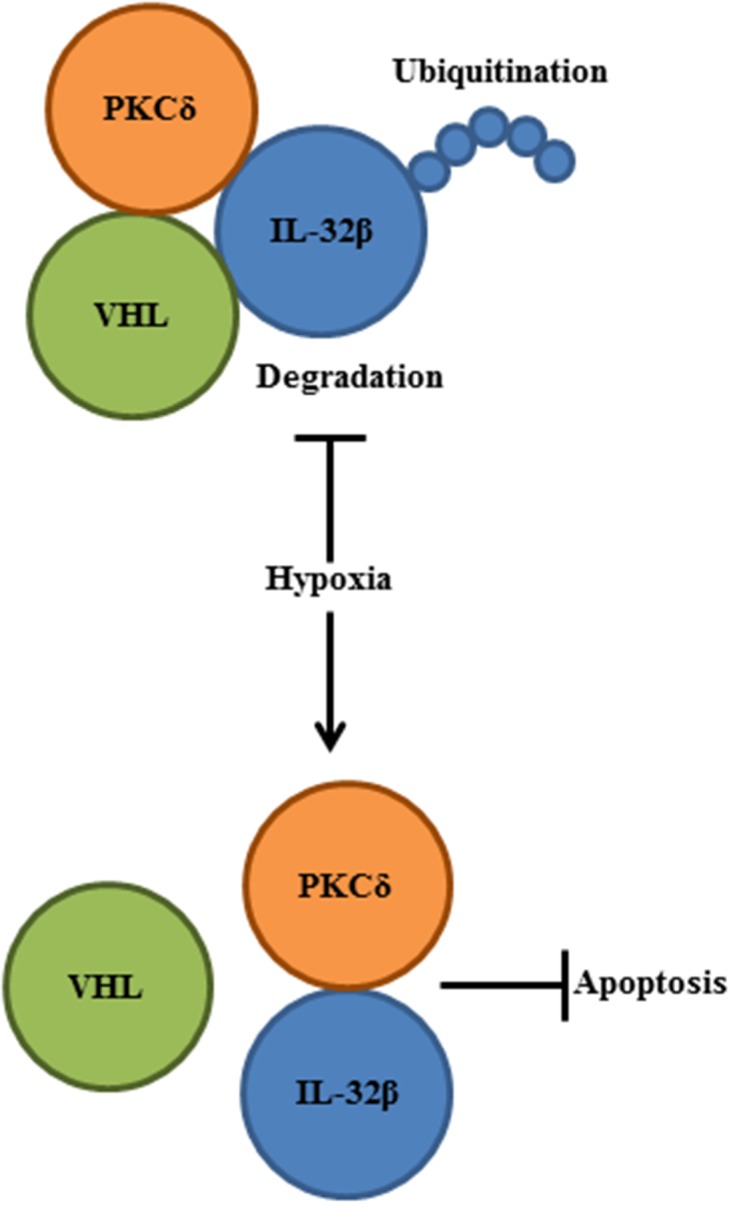
A schematic diagram summarizes IL-32β function in human ovarian cancer cells Hypoxia-induced ROS disrupted the interaction between IL-32β and VHL, leading to the IL-32β accumulation. The increased IL-32β bound to PKCδ in the oxidative stress condition and PKCδ-mediated apoptosis was inhibited.

Hypoxia-stabilized HIF-1α binds to hypoxia response elements, leading to the upregulation of several genes to promote survival under low-oxygen conditions. These genes code for enzymes that enhance glycolysis, which allows for ATP synthesis in an oxygen-independent manner [38, 39]. VEGF is a product of one of the HIF-1α-induced genes, which promotes angiogenesis and supplies nutrients and oxygen to the tumor mass [40]. In the present study, we identified one more protein (i.e., IL-32β) that is as urgently needed as HIF-1α under hypoxic and ROS conditions. IL-32β stability was regulated by the post-translational mechanism in SKOV3 and OVCAR8 human ovarian cancer cells. This cytokine was continuously synthesized and degraded by ubiquitination under normoxic conditions, but accumulated rapidly following exposure to low oxygen tension or ROS. Since the regulation of IL-32β in some of the ovarian tumor cells is energy-consuming, we infer that tumor cells urgently need IL-32β under the hypoxic and ROS conditions. We revealed that hypoxia- and ROS-stabilized IL-32β prevents PKCδ-mediated apoptosis, and this would rescue tumor cells from sudden death caused by environmental changes (Figure 7).

Various isoforms of IL-32 are produced by alternative splicing mechanism and there are conflicting observations of either an oncogenic or a tumor suppressive role for IL-32 [12, 41]. These contradictory results might originate from differences in the IL-32 isoform expressed in tumors analyzed. It is reported that IL-32γ and IL-32β induce caspase-8-dependent cell death in HEK293 cells whereas IL-32α does not, and that the treatment of alternative splicing inhibitor results in the predominant expression of IL-32γ splice variants and cell death in thyroid cancer cell lines [42]. On the other hand, IL-32β itself shows either an oncogenic or a tumor suppressive role. In this study, ROS-induced IL-32β reduced PKCδ-induced apoptosis, however, IL-32β transgenic mice have the increased cytotoxic T lymphocytes and NK cells activity, leads to the inhibition to tumor growth [15]. A plausible explanation about contrary IL-32β function could be that physiological environments such as ROS condition and change of immune system affects IL-32β role.

To find out which E3 ligases are responsible for IL-32β ubiquitination, the hypoxia- or ROS-related E3 ligases VHL, CHIP, and Keap1 [20, 29–32] were examined. Among these, only VHL could bind to and ubiquitinate IL-32β. Whereas VHL binding to HIF-1α is dependent on prolyl hydroxylation, the interaction between IL-32β and VHL was not dependent on prolyl hydroxylation of IL-32β. As with IL-32β, it is known that Aurora kinase A is also regulated by VHL in a prolyl hydroxylation independent manner [43]. We have therefore provided one more protein that is a VHL target, but for which binding is hydroxylation independent. On the other hand, Stoehr et al. showed that the inhibition of prolyl hydroxylation by DMOG stabilizes many proteins that have potential function under the hypoxic conditions [44]. Since DMOG treatment stabilized IL-32β (Figure 4D), the prolyl hydroxylation of IL-32β itself could stimulate its degradation under normoxic conditions, but it would not depend on VHL interaction, since we showed that the interaction between VHL and IL-32β was not dependent on IL-32β prolyl hydroxylation. Therefore, we can infer that IL-32β is degraded by two ways under normoxic conditions: prolyl hydroxylation-induced IL-32β degradation, and VHL-mediated degradation in a prolyl hydroxylation-independent manner. On the other hand, since prolyl hydroxylation-induced IL-32β degradation was not observed in OVCAR8 cells (Figure 4D, 4E), this process may need another factor that is absent in OVCAR8 cells.

It is known that PKCδ interacts with the β domain of VHL, but its overall protein levels are not affected by the interaction with VHL [45, 46]. The interaction of PKCδ with VHL retains PKCδ in the cytoplasm, which in turn impairs the association between PKCδ and the insulin-like growth factor 1 (IGF-I) receptor [37]. Therefore, besides the degradation of binding partners, another effect of the interaction with VHL is the limit of the localization of binding partners. In contrast to inactive PKCs, which are present mainly in the cytosol, activated PKCs are localized at the plasma membrane, the nucleus, and other subcellular compartments [47]. Hypoxic conditions in particular trigger the translocation of PKCδ to the endoplasmic reticulum or mitochondria, resulting in oxidative stress-induced apoptosis [24-26, 48]. Since the hypoxic stimulus translocates IL-32β to the mitochondria in breast cancer cells [14], IL-32β could inhibit PKCδ-mediated apoptosis. In turn, hypoxia- and ROS-stabilized IL-32β contributes to cancer cell survival. On the other hand, IL-32β interacts with PKCδ in the human monocytic cell line U-937, and its interaction shifts the immune reaction from an inflammatory to an anti-inflammatory one by enhancing anti-inflammatory cytokine IL-10 production and inhibiting the secretion of IL-1β and TNF-α [27, 28]. These findings suggest that IL-32β plays a favorable role in tumor cell survival by affecting the function of both tumor and immune cells.

In conclusion, hypoxia releases IL-32β from ubiquitination-mediated degradation. Subsequently, the increased IL-32β interacts with PKCδ and results in the inhibition of apoptosis, which can be induced by ROS-activated PKCδ. In turn, the hypoxia-induced IL-32β is beneficial for tumor cell survival under hypoxic and ROS environments. The limitation of our study is that the effect of IL-32β could not be validated using knockout mice, because this cytokine is not expressed in rodents. Nonetheless, the functions of IL-32β have been and continued to be unveiled with the use of various cancer models.

## MATERIALS AND METHODS

### Cell cultures and differentiation

The IGROV1, SKOV3 and OVCAR8 cells were maintained in RPMI-1640 medium (HyClone Laboratories, UT, USA) containing 10% heat-inactivated fetal bovine serum (FBS; HyClone Laboratories), whereas the HEK 293T cells were maintained in Dulbecco’s modified Eagle’s medium (HyClone Laboratories) containing 10% heat-inactivated FBS. All cells were incubated at 37°C in a humidified 5% CO_2_ incubator.

### Plasmids

The green fluorescent protein-tagged IL-32β (GFP-IL-32β), Flag-PKCδ, and Myc-IL-32 isoforms and IL-32β-expressing plasmids were obtained from Prof. Do-Young Yoon at Konkuk University. HA-Ub was obtained from Prof. Keun Il Kim at Sookmyung Women’s University. HA-VHL and HA-VHL19 were obtained from Prof. Jae Whan Song at Yeonsei University. The HA-VHL Y98N and W117R were purchased from Addgene (Cambridge, MA, USA). Truncated mutants of IL-32 were prepared by PCR and subcloned into the *Eco*RI-*Sal*I sites of pEGFPN2, or the *Eco*RI-*Xho*I sites of the Myc-pcDNA3.1 mammalian expression vector. VHL S111D was prepared by PCR and subcloned into the *Eco*RI-*Xho*I sites of the HA-pcDNA3 mammalian expression vector.

### Antibodies

The following antibodies were used: anti-IL-32 antibody (provided by Prof. Do-Young Yoon), anti-β-actin antibody, anti-HA antibody, anti-GFP antibody, anti-PKCδ antibody (Santa Cruz Biotechnology, Santa Cruz, CA, USA) anti-Flag antibody (Sigma Aldrich, St. Louis, MO, USA), anti-Myc-Tag antibody, and anti-PARP antibody (Cell Signaling Technology, Danvers, MA, USA).

### Chemicals

The following chemicals were obtained from commercial sources: CoCl_2_, CHX, MG-132, NAC, pepstatin A, H_2_O_2_, DMOG, and DFO (Sigma Aldrich).

### RNA interference and transfection

For the RNA interference assay, IL-32- and VHL-specific siRNA oligonucleotides were purchased from Samchully Pharmaceuticals (Seoul, Korea). The following sequences were used for the construction of the siRNAs: IL-32 siRNA forward 5'-GCUCUCUGUCAGAGCUCUU-3' and reverse 5'-AAGAGCUCUGACAGAGAGC-3'; and VHL siRNA forward 5'-CCACAGCUACCGAGUGUAUTT-3' and reverse 5'-AUACACUCGGUAGCUGUGGTT-3'. CHIP siRNA, Keap1 siRNA and PHD2 siRNA were purchased from Santa Cruz Biotechnology. PKCδ siRNA was purchased from Bioneer (Seoul, Korea). The cells were transfected with 10 nM of siRNA using RNAiMAX (Invitrogen, Carlsbad, CA, USA).

### Immunoblot analyses

The cell lysates were prepared after transfection with plasmids or siRNA and mixed with 5× sodium dodecyl sulfate (SDS) sample buffer. The mixed samples were heated at 99°C for 10 min and separated electrophoretically on a SDS-polyacrylamide gel. Subsequently, the proteins were transferred onto a 0.45-μm nitrocellulose membrane (GE Healthcare, Buckinghamshire, UK) for 2 h. The membrane was blocked for 30 min at room temperature with 5% skim milk (Invitrogen) and subsequently incubated with primary antibody overnight at 4°C. Then, the membrane was incubated with an anti-mouse or anti-rabbit IgG antibody conjugated to horseradish peroxidase (Assay Designs, Ann Arbor, MI, USA) at room temperature for 2 h. The proteins were visualized using an enhanced chemiluminescent substrate (Thermo Fisher Scientific, Logan, MA, USA) and analyzed using the LAS3000 luminescent image analyzer (Fuji Film, Tokyo, Japan). The protein bands were quantified using ImageJ software (National Institutes of Health, Bethesda, MD, USA).

### Immunoprecipitation assay

The HEK 293T and OVCAR8 cells were transfected with plasmids for 24 h and then treated with chemicals. The cell lysates were prepared using cell immunoprecipitation lysis buffer (50 mM Tris-HCl (pH 8.0), 150 mM NaCl, 5 mM ethylenediaminetetraacetic acid, 250 mM phenylmethylsulfonyl fluoride, 1% NP-40) containing a proteinase inhibitor cocktail. The cell lysates were incubated with antibody for 2 h at room temperature, and then protein-G agarose beads were added. The entire lysates were centrifuged after 2 h incubation, and then washed four times with washing buffer.

### RT-PCR

Total RNA samples were prepared from SKOV3 and OVCAR8 cells, using the extraction reagent RNAiso Plus (TaKaRa, Tokyo, Japan). The prepared total RNAs were reverse-transcribed using RevertAid reverse transcriptase (Thermo Fisher Scientific) at 42°C for 1 h. PCR was performed to amplify the mRNAs that encode IL-32 isoforms, β-actin, VEGF, VHL, CHIP, Keap1, and PHD2 using the following appropriate primer pairs: IL-32 forward 5'-ATGTGCTTCCCGAAGGTCCTC-3' and reverse 5'-TCATTTTGAGGATTGGGGTTC-3'; β-actin forward 5'-GTGGGGCGCCCCAGGCACCA-3' and reverse 5'-CTCCTTAATGTCACGCACGAT-3'; VEGF forward 5'-ATGAACTTTCTGCTGTCTTG-3' and reverse 5'-CCGCCTCGGCTTGTCACATCTGC-3'; VHL forward 5'-GAGGTCACCTTTGGCTCTTCA-3' and reverse 5'-CCAGATCTTCGTAGAGCGACC-3'; CHIP forward 5'-GTGATCACCCGGAACCCG-3' and reverse 5'-GCGCTTCTTCTTCGCGATTC-3'; Keap1 forward 5'-CATCCACCCTAAGGTCATGGA-3' and reverse 5'-GACAGGTTGAAGAACTCCTCC-3' and PHD2 forward 5'-GAAGGCAAAGCCCAGTTTGCT-3' and reverse 5'-CGTGCTCTCTCATCTGCATCAA-3'. Real-time PCR was performed with Maxima SYBR Green/ROX qPCR master mix (Thermo Fisher Scientific) to amplify the mRNAs that encode IL-32β, IL-32γ, and glyceraldehyde 3-phosphate dehydrogenase (GAPDH), using the following appropriate primer pairs: IL-32β forward 5'-CAGGGGAGATACCATGATCG-3' and reverse 5'-ACGGACTAATACGGCAACAGA-3'; IL-32γ forward 5'-TGACATGAAGAAGCTGAAGGC-3' and reverse 5'-CATGACCTTGTCACAAAAGCTC-3'; and GAPDH forward 5'-G TGTTCCTACCCCCAATGTGT-3' and reverse 5'-ATTGTCATACCAGGAAATGAGCTT-3'.

### Activity assay

The Caspase-Glo 3/7 assay (Promega, Madision, WI, USA) was carried out according to the manufacturer’s instruction. The assay is based on the release of amino-luciferin as a result of active caspase-3/7 cleaving the luminogenic substrate containing the DEVD amino acid sequence.

### Proliferation assay

Cell proliferation was evaluated using the CellTiter-Blue assay (Promega). Cells were seeded on 48-well plates and incubated for 24 h. Then, the cells were treated with CoCl_2_ for 24 h. After washing with PBS, diluted CellTiter-Blue reagent was added directly to each well and the plates were incubated at 37°C for 1 h. The reagent was transferred to 96-well plates and, the fluorescence was measured at 560/590 nm.

### *In vitro* migration assay

Cell migration was assessed in a 24-well plate Transwell system (Corning, Corning, MD, USA). Each 8-μm Transwell insert was seeded with 100 μL of SKOV3 or OVCAR8 cells in serum-free RPMI-1640 medium with or without CoCl_2_, and the lower chamber was filled with 500 μL RPMI-1640 medium containing 10% FBS with or without CoCl_2_. The cells were incubated for 24 or 48 h, respectively. Images of the membrane were taken in three random fields per chamber, and the total number of migrated cells was counted.

### Statistical analyses

Statistical analyses were performed by a paired Student’s t-test and one-way factorial analysis of variance. The Scheffe and Bonferroni tests were also performed. Values of *p < 0.05, **p < 0.01, and ***p < 0.001 were considered benchmarks of significant differences. Data are presented as the means ± standard deviation.

## Abbreviations

2OG: 2-oxoglutarate
CC-RCC: clear cell renal cell carcinoma
CHIP: carboxyl terminus of HSP70-interacting protein
CHX: cyclohexamide
CoCl2: cobalt chloride II
DAG: diacylglycerol
DFO: desferrioxamine
DMEM: Dulbecco’s modified Eagle’s medium
DMOG: dimethyloxaloylglycine
FBS: fetal bovine serum
FIH: factor inhibiting HIF
H2O2: hydrogen peroxide
HIF-1α: hypoxia-inducible factor-1alpha
HRE: hypoxia-response element
IGF-I: insulin-like growth factor 1
IL: interleukin
IP: Immunoprecipitation
Keap1: Kelch-like ECH-associated protein 1
NAC: N-acetyl-cysteine
NF-κB: nuclear factor kappa-light-chain-enhancer of activated B cells
NK cells: natural killer cells
Nrf-2: nuclear factor erythroid 2-related factor 2
PHD: prolyl hydroxylase
PKC: protein kinase C
ROS: reactive oxygen species
SD: standard deviations
SDS: sodium dodecyl sulfate
siRNA: small interfering RNA
STAT3: signal transducer and activator of transcription 3
TNFα: tumor necrosis factor alpha
VEGF: vascular endothelial growth factor
VHL: von Hippel-Lindau
